# High-density Grid Technology Aids in the Visualization of Purkinje Potentials in Fascicular Ventricular Tachycardia

**DOI:** 10.19102/icrm.2021.120121S

**Published:** 2021-01-15

**Authors:** Deepak Saluja, Geoffrey A. Rubin, Mark P. Abrams, Jeremy P. Berman, Elaine Y. Wan, Angelo Biviano, Hasan Garan

**Affiliations:** ^1^Electrophysiology Section, Division of Cardiology, Department of Medicine, Columbia University Vagelos College of Physicians and Surgeons, New York, NY, USA

**Keywords:** Purkinje potentials, right bundle branch block, ventricular tachycardia

A 71-year-old man with atrial fibrillation (AF), ischemic cardiomyopathy, and ventricular tachycardia (VT) presented having experienced implantable cardioverter-defibrillator (ICD) shocks. He had been previously treated with amiodarone and ICD placement for VT. His last episode of VT was several years prior to admission. About two months before admission, several appropriate shocks were recorded. Despite an increase in amiodarone, in subsequent weeks, he received several additional shocks with syncope. He was hospitalized and an unchanged ejection fraction of 35%, patent bypass grafts, and no new coronary disease were documented. He was then transferred to our institution.

In the electrophysiology laboratory, the baseline rhythm was AF with left bundle branch block (LBBB) and an H–V interval of 59 ms. Voltage mapping of the left ventricle (LV) endocardium with the Advisor™ HD Grid Mapping Catheter, Sensor Enabled™ revealed only a small area of scar in the anteroseptal LV. During mapping, incessant, repetitive episodes of VT no. 1 (VT1) occurred with an H–V interval of 79 ms **(Figure 1)**. Attempts at entrainment from the right ventricle repeatedly led to termination. Bundle-branch reentry VT was diagnosed and ablation of the right bundle led to the cessation of VT1.

Subsequently, repetitive episodes of a right-bundle, superior-axis morphology VT no. 2 (VT2) were seen. Intracardiac electrograms and a 12-lead electrocardiogram suggested an origin near the left posterior hemifascicle **(Figure 2)**. Entrainment attempts from this area led to termination. The Advisor™ HD Grid catheter was placed in this region **(Figure 3)**, demonstrating a fascicular potential of about 50 ms pre-QRS and retrograde Purkinje potentials. Ablation at this site resulted in termination of the repetitive VT2 salvos **(Video 1)**. The patient remained without further sustained VT at three months of follow-up.

This case suggests the Advisor™ HD Grid catheter is useful in rapidly identifying the presence and direction of conduction of fascicular potentials during the ablation of fascicular VT.

## Figures and Tables

**Figure 1:**
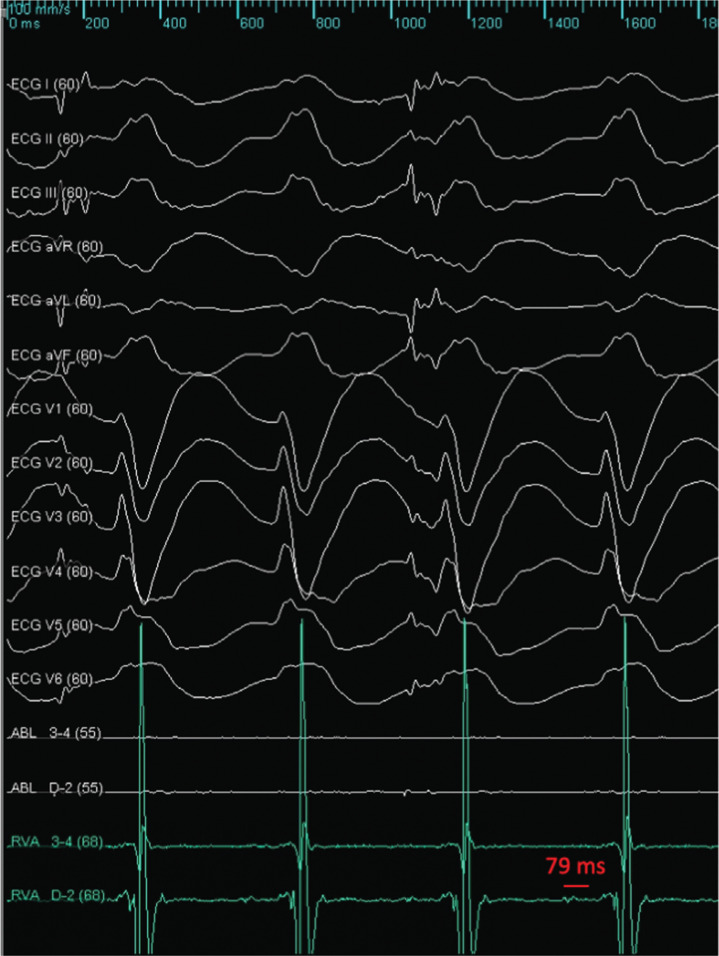
The baseline rhythm was AF with LBBB conduction and an H–V interval of 59 ms. VT1, repetitively initiated with catheter manipulation, had an H–V interval of 79 ms, suggestive of bundle-branch reentry VT. Attempts at entrainment led to termination. Ablation of the right bundle was performed with subsequent cessation of the VT. In this figure, the right ventricular catheter is in the His position.

**Figure 2:**
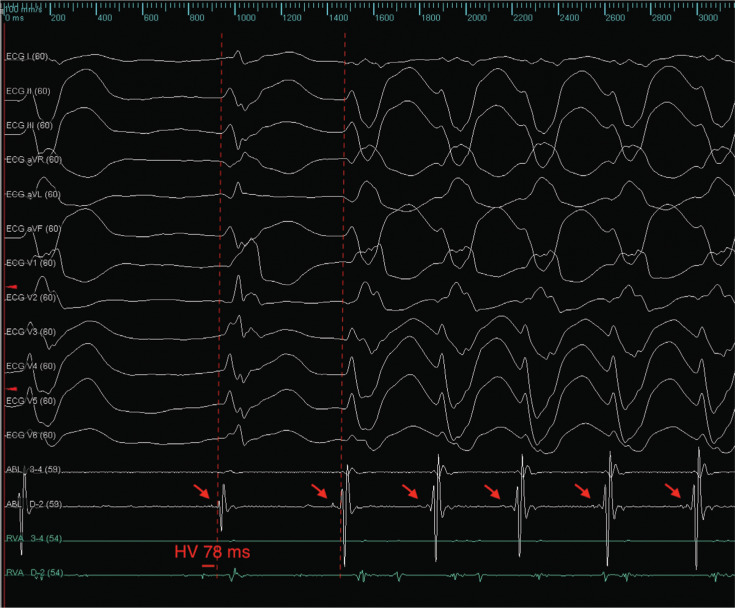
Repetitive episodes of VT were seen. The conducted QRS was a right-bundle type with a prolonged H–V interval after ablation of the right bundle. The ablation catheter is in the region of the left posterior hemifascicle. The potential QRS time was roughly 20 ms both in sinus rhythm and VT (arrows), suggesting the catheter was located at or downstream of a fascicular origin of the VT. In this figure, the right ventricular catheter is in the His position.

**Figure 3:**
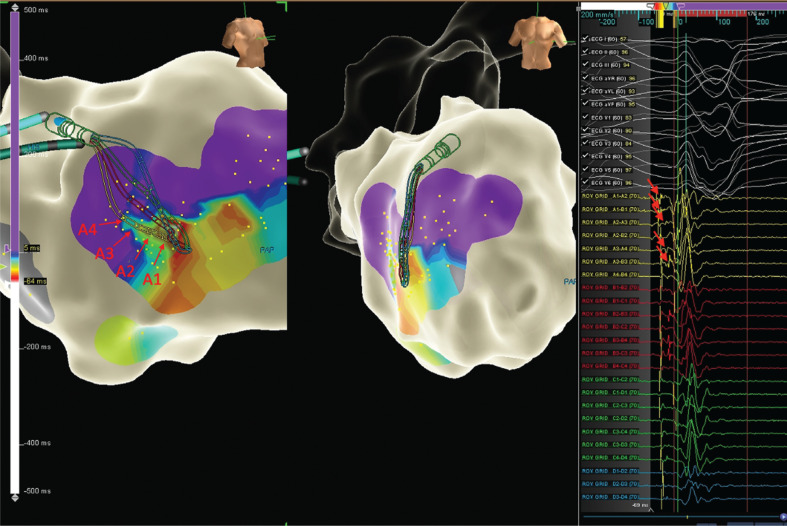
Advisor™ HD Grid signals showed the earliest Purkinje signals were at about 50 ms pre-QRS (A1–A2), with retrograde activation of Purkinje potentials evident (arrowheads).

**Video 1.** Ablation at the site of earliest activation causes termination of VT.

